# Plasma and urine biomarkers in acute viral hepatitis E

**DOI:** 10.1186/1477-5956-7-39

**Published:** 2009-10-27

**Authors:** Shikha Taneja, Somdutta Sen, Vijay K Gupta, Rakesh Aggarwal, Shahid Jameel

**Affiliations:** 1 Virology Group, International Centre for Genetic Engineering and Biotechnology, Aruna Asaf Ali Marg, New Delhi - 110067, India; 2 The Centre for genomic Applications, Okhla Industrial Area (Phase III), New Delhi - 110020, India; 3 Department of Gastroenterology, Army Hospital, Delhi Cantonment, New Delhi - 110010, India; 4 Department of Gastroenterology, Sanjay Gandhi Postgraduate Institute for Medical Sciences, Rae Bareilly Road, Lucknow - 226014, India

## Abstract

**Background:**

Hepatitis E, caused by the hepatitis E virus (HEV), is endemic to developing countries where it manifests as waterborne outbreaks and sporadic cases. Though generally self-limited with a low mortality rate, some cases progress to fulminant hepatic failure (FHF) with high mortality. With no identified predictive or diagnostic markers, the events leading to disease exacerbation are not known. Our aim is to use proteomic tools to identify biomarkers of acute and fulminant hepatitis E.

**Results:**

We analyzed proteins in the plasma and urine of hepatitis E patients and healthy controls by two-dimensional Differential Imaging Gel Electrophoresis (DIGE) and mass spectrometry, and identified over 30 proteins to be differentially expressed during acute hepatitis E. The levels of one plasma protein, transthyretin, and one urine protein, alpha-1-microglobulin (α1m), were then quantitated by enzyme immunoassay (EIA) in clinical samples from a larger group of patients and controls. The results showed decreased plasma transthyretin levels (p < 0.005) and increased urine α1m levels (p < 0.001) in acute hepatitis E patients, compared to healthy controls. Preliminary results also showed lower urine zinc alpha glycoprotein levels in fulminant hepatitis E compared to acute disease; this remains to be confirmed with more fulminant cases.

**Conclusion:**

Our results demonstrate the utility of characterizing plasma and urine proteomes for signatures of the host response to HEV infection. We predict that plasma transthyretin and urine α1m could be reliable biomarkers of acute hepatitis E. Besides the utility of this approach to biomarker discovery, proteome-level changes in human biofluids would also guide towards a better understanding of host-virus interaction and disease.

## Background

Hepatitis E is a significant public health problem in resource-limited regions of the world. The disease is caused by the hepatitis E virus (HEV), and appears as sporadic cases as well as large localized outbreaks of acute hepatitis [1]. The HEV is a non-enveloped virus with a ~7.2 kb positive-strand RNA genome and is classified in the family *Hepeviridae *[2]. Due to a possible zoonotic association [3-5], unusually high HEV seropositivity [6] and occasional sporadic disease [7] have also been observed in developed countries.

The disease is mild to moderate in severity with a 0.5 to 4% mortality rate [1]. However, it presents with increased severity and mortality approaching 15-25% in pregnant women, especially those infected in the third trimester of pregnancy [8]. While the disease is largely self-limited, a small fraction of sporadic cases progress to fulminant hepatic failure (FHF) with high mortality; this is also observed in infected pregnant women [1]. The obstetric and fetal outcomes are reported to be worse for pregnant women infected with HEV compared to other hepatitis viruses [9]. The events leading to FHF are not known and no predictive or diagnostic markers are available. Diagnostic tests routinely employed for HEV infection include evaluation of circulating IgM antibodies to viral components, occasional evaluation of viral RNA by reverse transcription polymerase chain reaction (RT-PCR) and the biochemical assessment of liver function [1]. None of these tests shed any light on disease prognosis. The availability of predictive biomarkers of disease progression would aid in identifying those sporadic cases that are more likely to develop severe disease, and would thus aid in patient care and management.

The outcome of an infection depends upon multiple factors, both pathogen-related and host-related. Changes in the host proteome following infection have been documented for a number of human pathogens [10]. Genomic and proteomic biomarkers are proving to be particularly useful in understanding disease progression, staging and response to therapy in chronic diseases and cancers [11,12]. Since disease progression also depends upon the host response to infection, examination of differential protein expression profiles from diseased versus healthy individuals is a good starting point to search for potential markers of disease progression.

Body fluids such as plasma and urine being in close contact with cells and tissues are good representatives of the host physiology. Proteomic methods such as two-dimensional gel electrophoresis (2DGE) and differential imaging gel electrophoresis (DIGE) followed by the mass spectrometric identification of proteins are being employed to compare signatures in body fluids from patients and controls [13,14]. We have applied these proteomic methods to compare plasma and urine from hepatitis E patients and healthy controls. Our results show changes in multiple proteins from both analytes, two of which were also validated in a larger sample set.

## Methods

### Subjects

Specimens were obtained from patients with jaundice at the Gastroenterology Outpatient Clinic or inpatients at the Army Base Hospital in New Delhi, India. Specimens were also obtained from healthy volunteers. Blood (5-6 ml) was collected in EDTA-coated vacutainers (Becton Dickinson), centrifuged at 800 × g for 5 min at 4°C. The plasma was collected and stored in aliquots at -70°C. It was tested for various viral hepatitis markers by enzyme immunoassays (EIA) as follows: HBsAg (Hepalisa, J Mitra and Co. Pvt. Ltd., New Delhi, India), anti-HCV (Hep-Chex-C, XCyton Diagnostic Pvt. Ltd., Bangalore, India), and IgM anti-HEV (HEV IgM ELISA, MP Biomedicals Asia Pacific Pvt. Ltd., Singapore). First morning mid-stream urine (~150 ml) was collected in an autoclaved bottle containing one protease inhibitor cocktail tablet (Roche, Germany), and stored at -70°C.

### Plasma fractionation and dye labelling

The plasma samples were first depleted of the six most abundant proteins using a MARS spun column (Agilent Technologies, USA) according to the manufacturer's protocol. Briefly, 15 μl of plasma was diluted in 185 μl of Buffer A, passed through a spin pre-column and then loaded onto the MARS column. The depleted fraction was collected as flow through and the bound proteins were eluted with Buffer B as recommended. The plasma depleted of 85-90% of albumin, immunoglobulin G (IgG), IgA, haptoglobin, anti-trypsin and transferrin was then cleaned up using the Clean-Up kit (GE Healthcare, UK) according to the manufacturer's protocol, and the protein content was estimated using the Bradford Reagent (Bio-Rad Laboratories). Pools of plasma specimens obtained from four patients (HEV pool) and four healthy volunteers (NOR pool) were prepared by mixing individual protein-depleted plasma in volumes that contained equal amounts of total protein. The protein pools were separately resuspended in DIGE buffer (8 M Urea, 4% CHAPS, 10 mM Tris-Cl, pH 8.5) to give a final protein concentration of 0.75 mg/ml. Cyanine dyes (Cy2, Cy3 or Cy5; GE Healthcare, UK) were reconstituted in anhydrous dimethylformamide and added to labelling reactions at a ratio of 400 pmoles of Cy Dye per 50 μg of protein. The reactions were kept for 30 min on ice in the dark and were terminated by the addition of 1 μl of 10 mM lysine for 10 min. The HEV and NOR pools were labelled with Cy5 and Cy3, respectively. An equal mixture of the two pools was also labelled with Cy2 and used as an internal control representing the total proteins. The labelled samples were either immediately fractionated further or were stored at -70°C.

### Multi Lectin Affinity Chromatography (MLAC)

Equal volumes of the samples labelled with Cy2, Cy3 and Cy5 dyes were mixed together and dialyzed against MLAC equilibration buffer (EB; 20 mM Tris-Cl, pH 7.4, 150 mM NaCl, 1 mM CaCl_2_, 1 mM MnSO_4_) overnight at 4°C with three changes of buffer. The MLAC column was prepared by mixing 700 μl packed bed volume each of Wheat Germ Agglutinin (WGA) agarose, Concavalin A (Con A) agarose and Jacalin agarose (Vector Laboratries, Inc., USA), and equilibrated with EB. The dialyzed proteins were applied to the column and allowed to bind. The flow through was recycled through the column once and then collected as the non-glycosylated fraction. The glycosylated proteins were then eluted using a displacer buffer (20 mM Tris-Cl, pH 7.4, 500 mM NaCl, 170 mM methyl α-D mannopyranoside, 270 mM galactose, 170 mM N-acetylglucosamine). The glycosylated and non-glycosylated plasma protein fractions thus obtained were either fractionated further using mixed cation-anion exchange chromatography (CAX) or were resolved using two-dimensional gel electrophoresis (2DGE).

### Mixed cation-anion exchange chromatography (CAX)

A mixed cation-anion exchange chromatography column was prepared with an equal mixture of S-Sepharose and Q-Sepharose (GE Healthcare, UK), and was equilibrated with 20 mM Tris-HCl pH 7.5. The glycosylated and non-glycosylated fractions were first dialysed against the CAX equilibration buffer overnight at 4°C with three changes and were separately applied to the column. After binding, the column was washed with two volumes of 20 mM Tris-HCl pH 7.5, and the bound proteins eluted with two volumes of a gradient of 100-600 mM NaCl in 20 mM Tris-HCl pH 7.5 (in steps of 100 mM NaCl). The fractions thus obtained were resolved by SDS-12%PAGE, the gels scanned on a Typhoon Imager (GE Healthcare, UK) and then silver stained to pick the bands for mass spectrometric analysis.

### Urine fractionation and dye labelling

Urine samples (50 ml) were centrifuged at 3,000 × g for 5 min at 4°C and the supernatant filtered through a Whatmann P3 paper. The filtrate was then dialyzed using a 3.5 kDa cut-off dialysis membrane (Pierce, USA) against MilliQ water overnight at 4°C with three changes. The dialysed sample was precipitated with 66% chilled acetone for 20 min on ice, followed by centrifugation at 12,000 × g for 20 min. The pellets were resuspended in a buffer containing 4 M Urea and 2% CHAPS, and the protein concentration estimated using the Bradford reagent (Bio-Rad Laboratories, USA). Labelling of the protein samples with Cy dyes was carried out as described for plasma proteins.

### Two-dimensional gel electrophoresis (2DGE)

The fractionated plasma proteins were precipitated with acetone at -20°C for 2 hr and then resuspended in rehydration buffer (8 M Urea, 2% CHAPS, 0.2% Bromophenol Blue, 20 mM DTT, 0.78% Pharmalyte). An IPGphor IEF unit and precast 13 cm, pH 3-10 NL IPG strips (GE Healthcare, UK) were used for the first dimension separation. The strips were rehydrated for 12 hr in rehydration buffer, 100 μg protein was loaded actively and resolved for a total focusing time of 32 KVhr. Each strip was then equilibrated with 5 ml equilibration buffer A (30% Glycerol, 6 M Urea, 50 mM Tris-HCl, pH 8.8, 2% SDS, 0.2% Bromophenol blue, 10 mg/ml dithiothreitol) on a rocker for 30 min at room temperature, followed by buffer B (30% Glycerol, 6 M Urea, 50 mM Tris-HCl, pH 8.8, 2% SDS, 0.2% Bromophenol blue, 25 mg/ml Iodoacetamide) for another 30 min at room temperature with rocking. The proteins were resolved in the second dimension at room temperature on SDS-10% polyacrylamide gels at 10 mA per gel for 30 min, followed by 20 mA per gel.

For the urine samples, 100 μg of labelled proteins were passively loaded during rehydration on 13 cm, pH 3.0-5.6 NL IPG strips for 12 hr at room temperature. The samples were focussed in the first dimension for 31 KVhr followed by strip treatment and second dimension as described above for plasma proteins.

The gels were scanned with an Ettan DIGE Scanner (GE Healthcare, UK) and analysed using the DeCyder 2D v6.5 software (GE Healthcare, UK). The intensity of each spot was normalized against the Cy2 labelled control. Only the spots that were significantly changed (= 1.5 fold increase or decrease) in the patients as compared to controls were selected for mass spectrometric analysis after silver staining of the gels.

### Mass spectrometry and protein identification

The gel plug was washed once with water, twice for 10 min each with vortexing in destaining solution (15 mM Potassium Ferricyanide, 50 mM Sodium Thiosulphate), twice in water for 15 min each, and then for 5 min with 100 μl of a solution containing 10 mM Ammonium Bicarbonate and 50% Acetonitrile (ACN). The proteins were reduced with 150 μl of 10 mM Dithiothreitol, 100 mM Ammonium Bicarbonate, 5% ACN for 1 hr at 55°C, followed by dehydration of the plug in 100 μl of 100% ACN for 20 min, and the proteins were alkylated with 100 μl of 50 mM Iodoacetamide in 100 mM Ammonium Bicarbonate for 30 min at room temperature, in the dark. The plugs were then washed with 100 μl of 100 mM Ammonium Bicarbonate for 10 min, followed by 100 μl of 100% ACN for 20 min. The liquid was removed and the plugs dried in a SpeedVac for 15 min. The proteins in gel plugs were then digested at 37°C with 150-200 ng trypsin in 50 mM Ammonium Bicarbonate for 16 hr. The extracted peptides were collected in the supernatant, the gel plugs treated once with 100 μl of 20 mM Ammonium Bicarbonate, twice with 100 μl of 1% Trifluoroacetic acid (TFA) in 50% ACN for 20 min, and once with 100 μl of 100% ACN for 20 min. All the supernatants were pooled together and concentrated in a SpeedVac to 10 μl. From this, 1 μl of the sample was mixed with 1 μl of α-Cyano-4-hydroxycinnamic acid (5 mg/ml in 50% acetonitrile, 0.1% TFA, Bruker Daltonics) matrix, and analyzed on the Bruker Ultraflex MALDI-TOF-TOF mass spectrometer with Flex Control v2.2, and processed using Flex analysis v2.2 software. The standard peptide mixture (a mixture of nine peptides from the m/z range of 757-3147) was used for external mass calibration, while self-degraded fragments of trypsin were used for internal calibration. Spectra were acquired for the mass-to-charge (*m/z*) range of 800-4000. All samples were analyzed in reflectron mode at laser power of 20-25%. Each spectrum was the sum of 500 laser shots with laser frequency of 50 Hz. The peak lists for MS were generated using Flex control version 2.2 and Flex analysis version 2.2 (Bruker Daltonics) using a proprietary "Top hat" base-line tool along with "SNAP" peak detection algorithm, which was set to a signal-to-noise ratio of 6, maximal number of peaks as 100 and quality factor threshold of 50. The MS/MS was smoothed and base line subtracted and the peptide mass list was searched against the MSDB database using the MASCOT search engine (BioTools v 2.2 software). MS/MS spectra were also processed using Flexanalysis v 2.2 software and searched using MASCOT. The mass spectrum data were submitted to an online database for protein identification (MASCOT version 2.1, ).

The search parameters were set as follows: Database - MASCOT; enzyme - trypsin; taxonomy - Homo sapiens; global modification of carbamidomethyl on cysteine; with variable modification of oxidation on methionine; no restrictions on protein mass; allowed up to 1 missed cleavage. The peptide tolerance was set at 100 ppm and MS/MS tolerance at ± 0.8-1.0 Da. For PMF, only those proteins with score >60 (*p *< 0.05) were accepted as identified, while for MS/MS individual ion cut off score was set as 39 or 41 (*p *< 0.05). The false discovery rate (FDR) was calculated using decoy database (MASCOT) and was 0.0% for most of the proteins (see Additional file 1). Xcorr was calculated for those proteins that were run on Thermo LCQ Deca nano LCMS and was searched in Sequest database. The Xcorr cut off was kept at 10.0 and the same data in .mgf format was also searched in MASCOT database and FDR calculated in decoy database. As to those peptides matched to multiple members of a protein family, the one with the highest score was reported. Most of the proteins identified were found to have more than two peptide matches in database however for few proteins with single peptide match, the sequence provided by the database was manually validated and those with reasonably good 'b and y' ion matches were considered (see Additional file 2, Additional file 3, Additional file 4 and Additional file 5). During protein identity validation due importance was also given to the theoretical molecular weight and isoelectric points of the proteins. The proteins with theoretical mass and isoelectric points approximately equal to the experimentally obtained values were accepted as successfully identified.

### Enzyme immunoassays (EIA)

For the quantitation of transthyretin and alpha-1-microglobulin (α1m) in body fluids, commercial EIA kits (Immunology Consultants Laboratory, Inc., USA) were used. The levels of transthyretin were assayed in plasma as per the manufacturer's protocol. To estimate α1m levels, urine was centrifuged at 3,000 × g for 5 min at 4°C. From this, 10 μL of the supernatant was diluted 100-fold with the diluent provided in the kit, and the assay carried out according to the manufacturer's protocols. The quantity of a specific protein in the test sample was interpolated from standard curves and corrected for sample dilution. Depending upon the viral markers, the samples were divided into five groups: Group 1, positive for HEV and negative for other viral hepatitis markers [HEV+/Other-] (n = 20); Group 2, positive for HEV and HBV [HEV+/HBV+] (n = 5); Group 3, negative for HEV but positive for HBV [HEV-/HBV+] (n = 5); Group 4, clinical diagnosis of viral hepatitis but negative for all viral markers [All-] (n = 4); Group 5, healthy controls (n = 20).

### Statistical analysis

Statistical significance of the data was evaluated with parametric as well as non-parametric methods. The former included the Student's t-test and the latter included the Mann-Whitney U test. These packages were part of the Past software [15]. P values of < 0.05 were considered significant. Receiver operating characteristic or ROC curve was generated using SPSS software (version 15.0). Area under the curve (AUC) was calculated and AUC values above 0.75 were considered.

## Results

### Plasma fractionation and analysis

**Figure 1:**
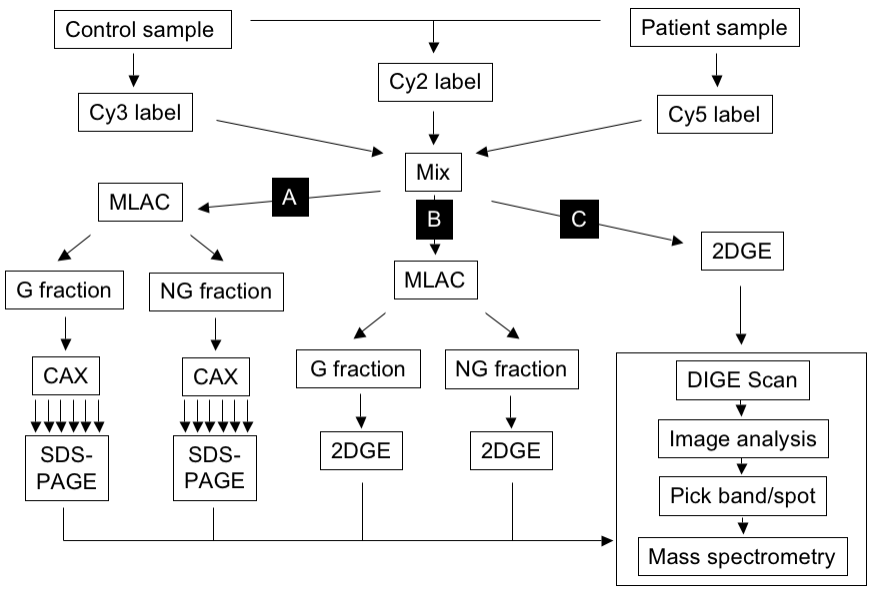
**Workflow for proteomic analysis**. Proteins in the plasma or urine of healthy controls and hepatitis E patients were labelled with Cy2, Cy3 or Cy5 dyes as described in Methods. The labelled samples were mixed and subjected to different fractionation strategies. In workflow A, the labelled samples were first subjected to Multi Lectin Affinity Chromatography (MLAC) to obtain fractions enriched in glycoproteins (G fraction) and non-glycoproteins (NG fraction). These fractions were then separately fractionated by mixed cation-anion exchange chromatography (CAX). The six fractions obtained by step salt elution were then analysed by SDS-PAGE. In workflow B, the labelled samples were subjected to MLAC and the enriched fractions were resolved by two-dimensional gel electrophoresis (2DGE). Workflows A and B were followed for plasma samples. Workflow C was followed for urine samples for which the labelled samples were directly fractionated by 2DGE. The differentially expressed spots were analysed with DeCyder 2D v6.5, the selected spots were cut out and the proteins identified by mass spectrometry.

Since the dynamic range of human plasma is high, we tried different proteomic workflows using pools of patient and control plasma labelled with cyanine dyes (Fig. 1). In workflow A, we separated labelled proteins into glycosylated and non-glycosylated fractions through multi-lectin affinity chromatography (MLAC) followed by a charge-based separation by mixed cation-anion exchange chromatography (CAX). These fractions were then analyzed by SDS-PAGE. In workflow B, the MLAC-derived glycosylated and non-glycosylated fractions were directly analyzed by two-dimensional gel electrophoresis (2DGE).

**Figure 2:**
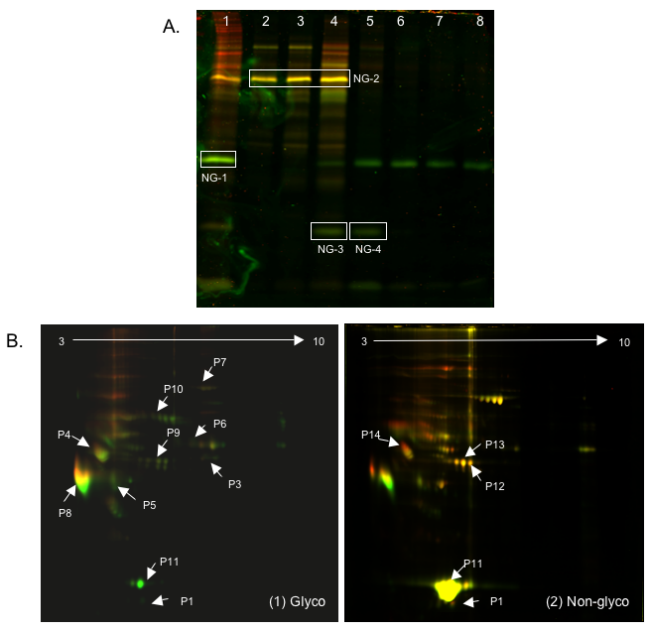
**Analysis of plasma fractions**. (A) Workflow A, NG fraction separated by CAX. Lanes: 1, NG fraction; 2, CAX flow-through fraction; 3-8, CAX eluates with 100, 200, 300, 400, 500 or 600 mM NaCl. The boxed bands NG-1, NG-2, NG-3 and NG-4 were identified by mass spectrometry. (B) DIGE images of (1) glycosylated and (2) non-glycosylated protein enriched fractions resolved by 2DGE. The proteins in spots P1 to P14 were identified by mass spectrometry and are shown in Table 1.

The main problem encountered with workflow A was the dilution of proteins at the end of the workflow, making it difficult to detect many proteins following SDS-PAGE and DIGE scans. From the non-glycosylated CAX fractions, we observed three proteins (NG-1, NG-3 and NG-4) to be downregulated in hepatitis E patients, and one protein (NG-2) that remained unchanged between patients and healthy controls (Fig. 2A). These were identified by mass spectrometry to be apolipoprotein A1 (NG-1), vitamin D binding protein (NG-2), and transthyretin or prealbumin (NG-3 and NG-4) (Table 1).

**Table 1:** Differentially expressed plasma proteins

**Identifier**	**Protein Name**	**Accession Number**	**Glyco/Non-Glyco**	**Fold-change**	**Acute phase reactant**
P1	Lipocalin Plasma retinol binding protein	AAF69622 1RBP	NG/G	-1.47(NG) +1.32(NG) -7.68(G) -6.09(G)	No ?(-)

P2	Transthyretin (prealbumin)	AAA61181	NG		Yes(-)

NG-3	- Transthyretin precursor - Interleukin 6 signal transducer isoform 1 precursor - Sequence 17 from patient EP1229047 unnamed protein product - HS422F24 chromosome 6 orf1	AAA61181 CAD48779 CAD48776 CA120537	NG		Yes(-)

NG-4	- Transthyretin (prealbumin) - Annexin A2 (Annexin II) (Lipocortin II) (Calpactin I Heavy Chain) - Possible J56 gene segment (fragment)	VBHU ANXA2_HUMAN	NG		Yes(-)

P3	Fibrinogen chain	FGHUB	G	+1.14 -1.59	Yes(+)

P4	- Alpha-2-HS glycoprotein - Cystatin C	WOHU AAF69649	G	+1.45 +1.02	Yes(-) No

P5	HP-Protein Haptoglobin	Q6NSB4 AAC27432	G	-2.27 -2.85	Yes (+)

P6	Beta-2 glycoprotein 1	1QUBA	G	-1.36 -2.58	No

P8	Alpha1 acid glycoprotein precursor 2 Alpha1 acid glycoprotein precursor 1	OMHU2 OMHU1 Q61B74	G	-2.15 -2.33	Yes (+)

P9	Fibrinogen	FGHUB	G	-0.82 -2.24	Yes (+)

P10	Hemopexin	CAA26382	G	-2.71 -2.78	Yes (+)

P11	Apolipoprotein A1	CAA00975	NG/G	-3.03(G) -7.90(G) -1.47(NG) -1.38(NG)	No

NG-1	Apolipoprotein A1	CAA00975	NG/G		

P12	Fibrinogen γ-A chain precursor Fibrinogen γ-B chain precursor	FGHUG FGHUGB	NG/G	+2.20(NG) +1.51(NG)	

P13	Tau Tubulin Kinase Hypothetical protein DKFZp686J1375	Q8IWY7 Q7Z3Q0	NG	+1.49 +1.43	No

P14			NG	+2.43 +2.94	

P15	Serum Amyloid P Precursor	YLHUP	G	-2.52	Yes (+)

NG-2	Vitamin D Binding Protein				

The differentially expressed plasma proteins from Figure 2 were analysed by DeCyder 2D v6.5 and identified by mass spectrometry. The table shows the spot intensity fold-changes in hepatitis E patients compared to healthy controls in two independent plasma pool experiments. The "+" and "-" values indicate increased or decreased expression, respectively, in patients compared to controls.

For workflow B, representative 2DGE patterns for the glycoprotein (Fig. 2B(1)) and non-glycoprotein fractions (Fig. 2B(2)) are shown. Various spots were marked using the DeCyder software and image analysis was carried out based on spot volume and intensity. Two independent sets of experiments with different patient and control pools were analysed. Representative gel images are shown for one set. In the glycoprotein fraction, of the 219 spots analyzed, 67 (30.6%) proteins were downregulated and 22 (10%) were upregulated in hepatitis E patients, while 130 (59.4%) remained unchanged. In the non-glycoprotein fraction, of the 102 spots analyzed, 34 (33.3%) proteins were downregulated and 33 (32.3%) were upregulated in hepatitis E patients, while 35 (34.3%) remained unchanged. The spots marked P1 to P15 (Fig. 2B) were excised and the proteins identified by mass spectrometry. The identity of these proteins and their average fold-changes in the plasma of hepatitis E patients versus controls in the two sets are shown in Table 1.

### Urine fractionation and analysis

**Figure 3:**
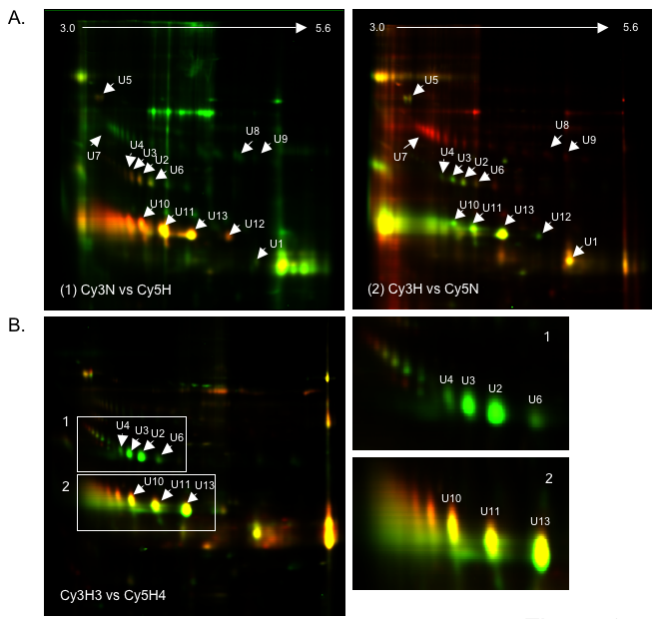
**Analysis of the urine proteome**. (A) In Workflow C, pooled urine samples were labelled and resolved by 2DGE. (1) DIGE image of proteins from pools of healthy normals and hepatitis E patients labelled with Cy3 and Cy5, respectively. (2) DIGE image of proteins from pools of hepatitis E patients and healthy normals labelled with Cy3 and Cy5, respectively. (B) DIGE image of urine proteins from acute mild hepatitis patient (H3) labelled with Cy3 and those from a case of fulminant hepatitis E (H4) labelled with Cy5. Box 1 shows proteins spots that were downregulated in fulminant hepatitis compared to acute mild infection, while Box 2 shows proteins that remained unchanged between the two forms of disease.

The protein content and dynamic range in urine is much lower than plasma. We therefore followed workflow C (Fig. 1) for analyzing urine samples from hepatitis E patients and controls. Various sets of samples were analyzed by 2DGE, DIGE scanning and quantitation using the DeCyder software, as described in Methods. Pools of 6 healthy and 9 hepatitis E patient urine samples were separately labelled with Cy3 and Cy5, respectively and analyzed according to workflow C. The DIGE image is shown in Fig. 3A (1). Of the 1177 spots subjected to image analysis, 38.3% proteins were upregulated and 22.5% proteins downregulated in acute hepatitis E, while 39.2% remained unchanged. A dye swap experiment was also carried out with the same pools, except that the hepatitis E patient samples were now labelled with Cy3 and the normal samples with Cy5. The DIGE image for this is shown in Fig. 3A (2). For 765 spots, image analysis showed comparable results with 33.5% proteins upregulated and 30.2% proteins downregulated in acute hepatitis E, and 36.3% remaining unchanged. There was good concordance for individual proteins as well; this is presented in Table 2. Our analyses also showed that samples collected more than 15 days after the onset of symptoms appeared similar to healthy control samples (not shown). In this set, only 6% proteins were upregulated, 2% were downregulated and 92% remained unchanged. Thirteen differentially expressed protein spots (U1 to U13) were picked (Fig. 3A) and identified by mass spectrometry. A list of these proteins and their fold changes during acute hepatitis E is shown in Table 2.

**Table 2:** Differentially expressed urine proteins

**Identifier**	**Protein Name**	**Accession Number**	**Fold Change Expt 1(HEV Cy5; CON Cy3)**	**Fold Change Expt 2 (HEV Cy3; CON Cy5)**
U1	Prostaglandin D2 synthase 21 kDa (Brain)	Q5SQ09	+2.16	-1.43

U2	- Zinc alpha 2 glycoprotein chain A - Alpha 1 type I collagen, preprotein	1ZAGA Q8N473_HUM	+4.97	-3.14

U3	Zinc alpha 2 glycoprotein chain A	1ZAGA	+6.58	-3.74

U4	Zinc alpha 2 glycoprotein chain A	1ZAGA	+6.98	-2.94

U5	KIAA0216 splice variant 1	Q5W9G1_HUM	+4.40	-1.85

U6	- Membrane associated guanylate kinase 2 - ODF2 protein	AAC05370 Q5BJF6_HUM	+3.33	-1.75

U7	FLJ00133 fragment	Q8TERO_HUM	+9.08	-1.83

U8	Serum albumin	AAA64922	-4.06	+8.67

U9	Serum albumin	AAA64922	-2.52	+6.67

U10	N2B-Titin Isoform RasGAP-activating-like protein 1 variant	CAD12455	+9.42	-3.81

U11	Alpha-1 microglobulin/inter alpha trypsin inhibitor precursor	HCHU	+6.01	-3.63

U12	Alpha-1 microglobulin/inter alpha trypsin inhibitor precursor	HCHU	+7.59	-4.21

U13	Alpha-1 microglobulin/inter alpha trypsin inhibitor precursor	HCHU	+6.22	-2.81

The differentially expressed urine proteins from Figure 3 were analysed by DeCyder 2D v6.5 and identified by mass spectrometry. The table shows the spot intensity fold-changes in hepatitis E patients (HEV) compared to healthy controls (CON). A dye swap experiment is also shown. The "+" and "-" values indicate increased or decreased expression, respectively, in patients compared to controls.

We also compared the urine proteins from a patient with acute mild infection (H3) labelled with Cy3 to those from a patient with fulminant hepatitis E (H4) labelled with Cy5 (Fig. 3B). Of the 725 spots detected, 80 (11%) were upregulated and 101 (14%) were downregulated in fulminant compared to mild hepatitis E infection, while 544 (75%) remained unchanged. The protein spots in box 1 (Fig. 3B) correspond to proteins U2, U3 and U4, which were identified as different isoforms of the Zinc Alpha Glycoprotein (ZAG), and U6 identified as the membrane-associated guanylate kinase 2, ODF2 protein (Table 2). The different isoforms of ZAG increased by 40 to 50-fold in acute hepatitis E compared to healthy controls, but decreased by 4 to 6-fold during the fulminant stage of disease, compared to the acute stage. Similarly, the membrane-associated guanylate kinase 2 and ODF2 protein increased about 17-fold during acute hepatitis E, but declined by about 3-fold during fulminant disease. Since this analysis depends upon a single fulminant hepatitis E sample, these changes require confirmation with more fulminant cases.

### Validation of proteomic results by EIA

The proteomic analyses used a limited number of samples from hepatitis E patients and controls. It was therefore important to independently validate representative markers in a larger sample set. Further, since only acute hepatitis E patients were compared to healthy controls in the proteomic analyses, it was not clear whether the identified proteins were differentially expressed in all types of viral hepatitis or were markers specific to hepatitis E. We looked at one marker each from plasma and urine, for which enzyme immunoassays (EIA) were commercially available. This included plasma transthyretin and urine α1m. Patient samples were divided into five groups depending upon their viral hepatitis markers, as described in Methods.

**Figure 4:**
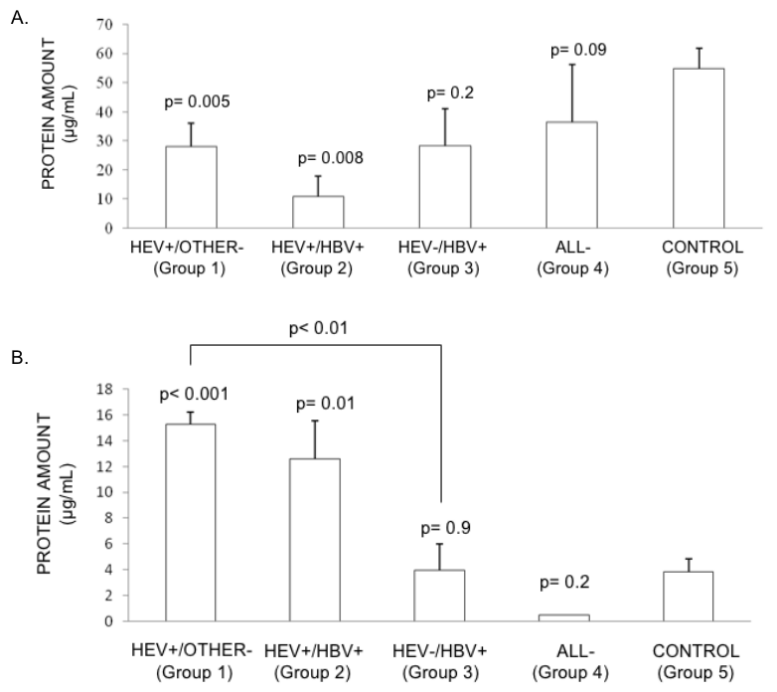
**Validation of representative biomarkers by EIA**. The EIAs were carried out as described in Methods. (A) Plasma levels of transthyretin were estimated for various groups of hepatitis patients and controls. The p values shown are based on the Mann-Whitney test; the significance remains the same with the Student's t-test. (B) The levels of alpha-1-microglobulin in urine were estimated for various groups of hepatitis patients and controls. The p-values shown are based on both parametric (Student's t test) and non-parametric (Mann-Whitney U test) tests. All p values shown are relative to Group 5, except in (B) in which Groups 1 and 3 were also compared.

The plasma transthyretin levels were significantly reduced in HEV mono-infected and HEV/HBV co-infected patients compared to healthy controls (Fig. 4A). However, patients with HBV infection (Group 3) or those with a clinical diagnosis of viral hepatitis without the serological markers (Group 4) showed no significant association, possibly due to the low numbers of samples (Fig. 4A). The urine α1m levels were significantly higher in HEV mono-infected and HEV/HBV co-infected patients compared to healthy controls (Fig. 4B). There were no significant changes in the urine α1m levels in patients in the other groups compared to controls. The HEV infected patients (Group 1) also showed significantly higher levels of urine α1m when compared to HBV infected patients (Group 3) (Fig. 4B). Area under the curve (AUC) for alpha 1 microglobulin assay was found to be 0.893 (95% confidence intervals = 0.795-0.990) and that for transthyretin assay was 0.763 (95% confidence intervals = 0.591-0.934). These analyses indicate that these two markers in particular urine α1m may be selective for acute hepatitis E infection.

## Discussion

Proteome analysis is a powerful way to study the pathophysiology of chronic and infectious human diseases [10]. Comparisons of the proteomes of tissues and biofluids from diseased and healthy persons have identified potential biomarkers to predict disease stages, clinical outcomes and response to therapy [12]. For example, haptoglobin and its glycosylated forms have been identified as potential biomarkers for non-small cell lung cancer and hepatocellular carcinoma [16,17], calcitonin for thyroid cancers [18], and gastrin for gastric and colorectal cancers [19]. A number of studies have also addressed disease biomarkers, such as decreased α1m in IgA nephropathy [20] and hepatocyte growth factor for liver dysregulation [21]. Reduced transferrin and increased alpha-2-macroglobulin in HBV carriers might suggest active liver disease [22]. Recently, complement C3a was predicted to be a candidate marker of chronic hepatitis C and HCV related hepatocellular carcinoma [23]. Some novel proteins associated with HCV-induced fibrosis have also been identified as inter-alpha trypsin inhibitor heavy chain 4 (ITIH4) fragments, complement factor H-related protein 1, CD5L, Apo L1, β2GPI, and thioester-cleaved products of alpha-2-macroglobulin [24]. So far there are no reports on markers of hepatitis E disease.

We report here the application of DIGE, a fluorescent protein labelling and gel electrophoresis platform to reproducibly fractionate and identify differentially expressed protein patterns in the plasma and urine of hepatitis E patients. One set of proteins that showed differential expression was the acute phase proteins (APP), whose plasma concentrations either increase (positive APP) or decrease (negative APP) during inflammation [25]. Though the APPs have different functions, they are primarily related to host defence. As liver is the major site for production of these proteins, any infection or dysfunction of the liver leads to changes in plasma APP levels. In acute hepatitis E we found the plasma levels of many positive APPs like haptoglobin, hemopexin, serum amyloid P precursor and alpha-1 acid glycoprotein to decrease. The haptoglobin-hemopexin system is the major vehicle for heme transport in the plasma and protects from the loss of heme-bound iron. It protects from oxidative stress and increases during an inflammatory response [26,27]. The plasma levels of alpha-1 acid glycoprotein (or orosomucoid) increase in response to systemic tissue injury, inflammation or infection, and these changes have been correlated to increases in hepatic synthesis. It functions as an immunomodulator [28] and its downregulation during acute hepatitis E is likely to compromise the innate immune response. The negative APP alpha 2 HS glycoprotein (or fetuin), whose plasma levels increase in acute hepatitis E patients, is anti-inflammatory [29]. We have previously shown that the ORF3 protein of HEV downregulates the transcription of multiple positive APP genes through the STAT3 transcription factor [30]. Thus, by actively downregulating the expression of plasma proteins involved in innate immune responses, HEV attenuates inflammatory responses and creates a favourable environment for viral replication and establishment of infection.

Transthyretin or pre-albumin is another negative APP whose levels are a reliable indicator of acute liver disease [31]. This was found to be differentially expressed in the plasma of hepatitis E patients. In patients with HEV infection, whether with or without HBV co-infection, the plasma transthyretin levels were significantly lower than those in healthy controls (Fig. 4A).

We also compared the urine proteomes of hepatitis E patients and healthy controls. Urine is an easily available non-invasive analyte, which has been used extensively for the diagnosis and monitoring of both renal and systemic diseases [32]. Being a filtrate of blood, urine also carries protein components analogous to plasma, but with a reduced diversity and dynamic range. The levels of α1m were significantly higher in the urine of hepatitis E patients compared to healthy controls and patients with other forms of acute viral hepatitis. Alpha-1-microglobulin is a 31-kDa glycoprotein, which is involved in defending tissues against oxidation by heme, kynurenin and reactive oxygen species [33-35], and also functions as an immunosuppressor [35,36]. Elevated levels of α1m have also been associated with renal tubular disorders [37]. In earlier studies, the ORF3 protein of HEV was shown to expedite the secretion of α1m from infected hepatocytes [38].

The levels of prostaglandin D2 synthase (PGD2S) were also found to increase in the urine of hepatitis E patients. It is a lipocalin involved in the transport of bile pigments, retinoids, thyroid hormone, bilirubin and biliverdin, as also a vasodilator produced by inflammatory cells (mast cells and macrophages), which suppresses the induction of inducible nitric oxide (NO) synthase, resulting in reduced inflammation [39]. Though PGD2S levels in serum and urine have been investigated as an indicator of renal dysfunction [40,41], there are no reports linking it to viral hepatitis. Our proteomic observation of increased levels of PGD2S in the urine of hepatitis E patients awaits further validation.

While a majority of HEV infections present as self-limited acute hepatitis, occasional cases present as FHF. The levels of zinc alpha glycoprotein (ZAG), membrane associated guanylate kinase 2 and ODF2 were significantly lower in the urine of a fulminant hepatitis E patient compared to a patient with mild hepatitis due to HEV. Interestingly, when compared to healthy persons, the levels of these proteins are still higher in hepatitis E patients, but as the disease takes a more severe fulminant form, they are partially cleared from the urine. Though the exact role of ZAG with respect to viral disease has not been shown, it plays a role in lipolysis in adipocytes and is involved in the regulation of body weight [42-44], besides being a soluble non-conventional major histocompatibility complex class I molecule [45]. Whether ZAG, membrane associated guanylate kinase 2 and ODF2 turn out to be potential markers of fulminant hepatitis E, will await confirmation from the analysis of more fulminant cases.

We present here the first study to employ proteomic analysis tools to look for potential biomarkers of hepatitis E disease. Our results show both plasma and urine to be analytes that are readily amenable to a differential protein labelling method such as DIGE followed by fractionation and mass spectrometric analysis. In a limited study we also show validation of two potential biomarkers by EIA. We have also identified a number of host proteins that are differentially expressed during HEV infection, which would help better understand the biology of host-pathogen interaction.

## Conclusion

This study demonstrated that plasma and urine proteomes of hepatitis E patients were amenable to differential labelling and proteomic analysis methods to discover signatures of the host response to viral infection. Besides identifying a number of differentially expressed proteins in the plasma and urine of hepatitis E patients, this study also showed that plasma transthyretin and urine α1m could be reliable biomarkers of acute hepatitis E. Initial results have also indicated urine proteome differences in patients with mild and fulminant hepatitis E. Besides the utility of this approach to biomarker discovery, proteome-level changes in the biofluids of infected persons would also guide towards a better understanding of host-virus interaction and hepatitis E pathogenesis.

## Competing interests

The authors declare that they have no competing interests.

## Authors' contributions

ST and SJ planned the experiments; ST carried out the experiments; SS did the mass spectrometric analyses; VKG and RA provided the clinical samples; RA helped with the statistical analyses; ST and SJ wrote the manuscript. All authors read and approved the final manuscript.

## Supplementary Material

Additional file 1 **The table shows protein identification and database search parameters for the plasma and urine proteins.** Click here for file

Additional file 2 **Spectra for single peptide assignments (TIFF files).** These show the spectra for the protein identities with single peptide matches in the database. Click here for file

Additional file 3 **Spectra for single peptide assignments (TIFF files).** These show the spectra for the protein identities with single peptide matches in the database. Click here for file

Additional file 4 **Spectra for single peptide assignments (TIFF files).** These show the spectra for the protein identities with single peptide matches in the database. Click here for file

Additional file 5 **Spectra for single peptide assignments (TIFF files).** These show the spectra for the protein identities with single peptide matches in the database. Click here for file
