# Supplementary figures and images for: Plasma and urine biomarkers in acute viral hepatitis E
Source: Proteome Sci. 2009 Oct 27;7:39. doi: 10.1186/1477-5956-7-39 (PMC2773234; doi:10.1186/1477-5956-7-39)

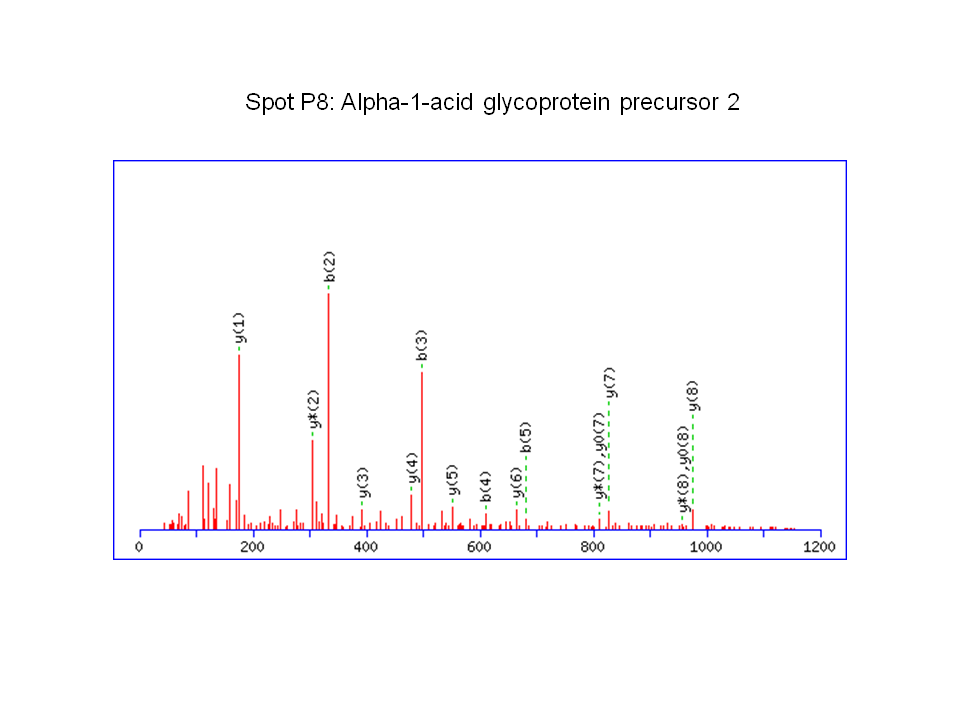

Supplement: Additional file 2 — Spectra for single peptide assignments (TIFF files). These show the spectra for the protein identities with single peptide matches in the database. [file 1477-5956-7-39-S2.TIFF]

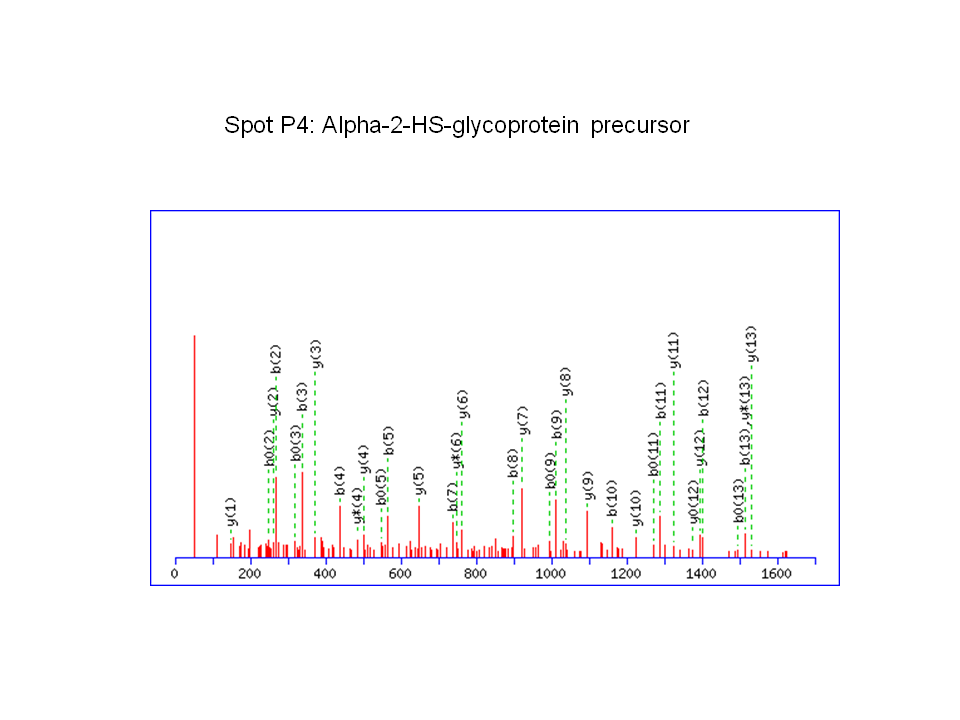

Supplement: Additional file 3 — Spectra for single peptide assignments (TIFF files). These show the spectra for the protein identities with single peptide matches in the database. [file 1477-5956-7-39-S3.TIFF]

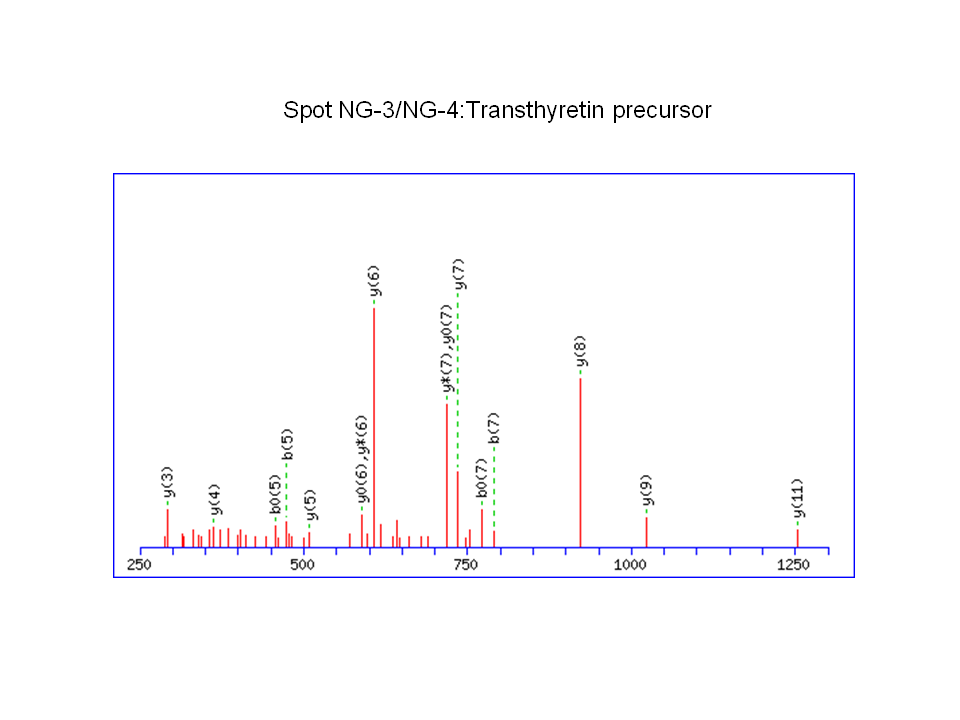

Supplement: Additional file 4 — Spectra for single peptide assignments (TIFF files). These show the spectra for the protein identities with single peptide matches in the database. [file 1477-5956-7-39-S4.TIFF]

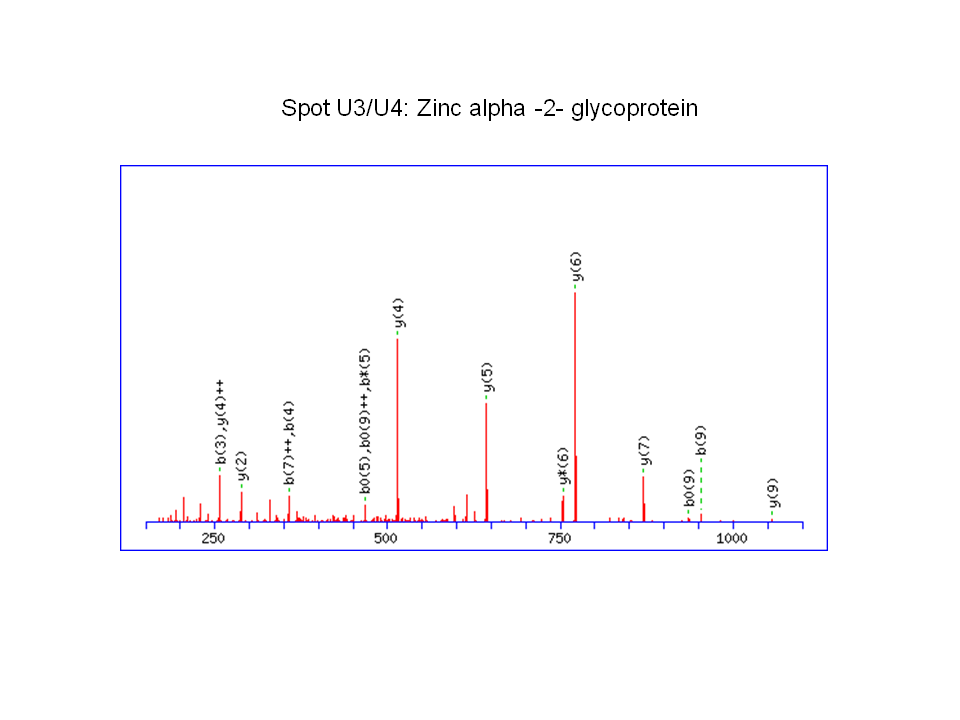

Supplement: Additional file 5 — Spectra for single peptide assignments (TIFF files). These show the spectra for the protein identities with single peptide matches in the database. [file 1477-5956-7-39-S5.TIFF]
